# Chronological Change of Resistance to β-Lactams in Salmonella enterica serovar Infantis Isolated from Broilers in Japan

**DOI:** 10.3389/fmicb.2013.00113

**Published:** 2013-05-21

**Authors:** Takehisa Chuma, Daisuke Miyasako, Hesham Dahshan, Tomoko Takayama, Yuko Nakamoto, Francis Shahada, Masato Akiba, Karoku Okamoto

**Affiliations:** ^1^Laboratory of Veterinary Public Health, Department of Veterinary Medicine, Joint Faculty of Veterinary Medicine, Kagoshima UniversityKagoshima, Japan; ^2^Department of Veterinary Public Health, Faculty of Veterinary Medicine, Zagazig UniversityZagazig, Egypt; ^3^Safety Research Team, National Institute of Animal HealthTsukuba, Japan

**Keywords:** antimicrobial resistance, β-lactamase gene, broiler, extended-spectrum cephalosporin, *Salmonella enterica* serovar Infantis, plasmids

## Abstract

Epidemiologic surveillance study was conducted in southern Japan to determine the antimicrobial resistance phenotypes and characterize the β-lactamase genes and the plasmids harboring these genes in *Salmonella enterica* serovar Infantis (*S*. Infantis) isolates from broilers. Between January, 2007 and December, 2008, a total of 1,472 fecal samples were collected and examined at the Laboratory of Veterinary Public Health, Kagoshima University, Japan. In 93 (6.3%) isolates recovered, 33 (35.5%) isolates showed resistance to cefotaxime, an extended-spectrum cephalosporin (ESC), conferred by TEM-20, TEM-52 and CTX-M-25 extended-spectrum β-lactamases (ESBLs). In addition to ESC-resistance, eight (8.6%) isolates exhibited resistance to cefoxitin mediated by CMY-2 AmpC β-lactamase. Plasmid analysis and polymerase chain reaction replicon typing revealed the *bla*_TEM-20_ and *bla*_CMY-2_ genes were associated with IncP plasmids, *bla*_TEM-52_ was linked with a non-typable plasmid and *bla*_CTX-M-25_ was carried by an IncA/C plasmid. Non-β-lactam resistance to streptomycin, sulfamethoxazole, and oxytetracycline encoded by the *aadA1*, *sul1*, and *tet*(A) genes, respectively, was found in 86 (92.5%) isolates. Resistance to kanamycin and ofloxacin was exhibited in 12 (12.9%) and 11 (11.8%) isolates, respectively, the former was mediated by *aphA1-Iab*. These data indicate that *S.* Infantis isolates producing ESBLs and AmpC β-lactamase have spread among broiler farms in Japan. These data demonstrated that the incidence of ESC-resistant S. Infantis carrying *bla*_TEM-52_ remarkably increased and *S.* Infantis strains harboring *bla*_CMY-2_, *bla*_TEM-20_, or* bla*_CTX-M-25_ genes emerged from broilers in Japan for the first time in 2007 and 2008.

## INTRODUCTION

Non-typhoidal *Salmonella enterica* serovars (*Salmonella*) are a major cause of bacterial food-borne diseases world-wide (Majowicz et al., 2010). The food poisoning statistics in Japan show that bacterial food poisoning patients during the year 2008 numbered at 10,331 and *Salmonella* were the leading etiological agents accounting for 24.7% of the cases [http://idsc.nih.go.jp/iasr/29/342/graph/t3421.gif]. Poultry products are important vehicles in the transmission, and have been incriminated in several *Salmonella* outbreaks (Kimura et al., 2004; Chittick et al., 2006). Since the late 1990s, *Salmonella enterica* serovar Infantis (*S*. Infantis) has been the commonest serovar of *Salmonella* isolated from both broiler flocks and retail chicken meat in Japan (Asai et al., 2007b; Iwabuchi et al., 2011).

The emergence of multidrug-resistant *Salmonella* has become a serious global health problem because antimicrobial treatment is lifesaving for invasive infections, particularly in neonates of <1 year of age. Preventive antimicrobial treatment is also generally given to patients suffering from immunosuppressive or other predisposing conditions (Hohmann, 2001). The options of first-line therapy for *Salmonella* infection include ampicillin (AMP), sulfamethoxazole-trimethoprim, fluoroquinolones, and extended-spectrum cephalosporins (ESCs). ESCs are among the preferred drugs because of resistance to the other aforementioned drugs is relatively frequent in *Salmonella* isolates (Hohmann, 2001). At present, ESC-resistance in *Salmonella* is mainly attributed to the acquisition of plasmid-mediated extended-spectrum β-lactamases (ESBLs) and AmpC β-lactamases (Bonnet, 2004; Arlet et al., 2006).

*Salmonella* carrying the *bla*_CMY-2_ gene were recently recovered from bovine and porcine salmonellosis cases (Dahshan et al., 2010; Sugawara et al., 2011). Besides, *S. *Infantis isolates harboring the *bla*_TEM-52_ gene were reported from broilers for the first time in the year 2004 (Shahada et al., 2010a). Thus, a major concern has been increased prevalence of resistance to ESCs noted in *S*. Infantis isolates. This study was conducted to determine the antimicrobial resistance phenotypes and characterize the β-lactamase genes and the plasmids harboring these genes in *S*. Infantis isolated from broiler flocks.

## MATERIALS AND METHODS

### BACTERIAL ISOLATES

Between January, 2007 and December, 2008, a total of 1,472 cecal specimens derived from 92 broiler flocks (ca. 10,000 birds per flock) were collected from a poultry processing plant located in the southern part of Japan. Usually, 16 samples per flock were randomly selected fortnightly. The isolation, identification and serotyping of S. Infantis isolates were performed at the Laboratory of Veterinary Public Health, Kagoshima University, Japan as previously described (Shahada et al., 2008).

### DETERMINATION OF MINIMUM INHIBITORY CONCENTRATIONS

Antimicrobial susceptibility testing was assayed by the agar dilution method on Mueller-Hinton (MH) agar (Oxoid Ltd., Basingstoke, Hampshire, England) plates according to the National Committee for Clinical and Laboratory Standards guidelines (National Committee for Clinical Laboratory Standards, 2001). *S*. Infantis isolates were tested for sensitivities to AMP, cefotaxime (CTX), cefoxitin (FOX), chloramphenicol (CHL), streptomycin (STR), sulfamethoxazole (SUL), oxytetracycline (TET), kanamycin (KAN), and ofloxacin (OFX). The MIC range was set at 0.125–512 μg/ml for all tested antimicrobial agents. The MIC breakpoints were interpreted according to the new criteria established by the Clinical and Laboratory Standards Institute (2012). *Escherichia coli* (*E. coli*) ATCC 25922 and *Staphylococcus aureus* ATCC29213 were used as quality control strains.

### DOUBLE-DISK SYNERGY ASSAY

The double-disk synergy testing was conducted to screen for ESBLs and AmpC β-lactamases as previously described (Shahada et al., 2010a). This test was performed as a standard Kirby-Bauer disk diffusion assay on MH agar (Oxoid) plates.

### DETECTION OF RESISTANCE DETERMINANTS

All DNA templates were prepared using the InstaGene Matrix kit (Bio-Rad Laboratories, Hercules, CA, USA). Detection of resistance genes was performed by polymerase chain reaction (PCR). The amplification reactions were carried out using primers and conditions as previously described (Dahshan et al., 2010; Shahada et al., 2010b). Briefly, the targets were as follows: *bla*_TEM_, *bla*_OXA_, *bla*_PSE,_ *bla*_SHV_, *bla*_CTX-M-1_ group, *bla*_CTX-M-2_ group, *bla*_CTX-M-25_, and *Toho-1* encoding for penicillinases; *bla*_CIT_, *bla*_CMY-2_, *bla*_DHA_, *bla*_FOX_, *bla*_MOX_, *bla*_ACC_, and *bla*_EBC_ encoding for cephalosporinases; *tet*(A), *tet*(B), and *tet*(G) mediating tetracycline efflux proteins; *aadA1* and *aadA2* encoding for resistance to STR and spectinomycin; *sul1* conferring resistance to SUL; and *aphA1-Iab* encoding for resistance to KAN.

When β-lactamase-encoding genes were positive following PCR amplification, obtained products were directly sequenced using a BioDye Terminator version 3.1 Ready Reaction sequencing kit and ABI 3100 automated DNA sequencer (Applied Biosystems, Foster City, CA, USA). The DNA alignments and deduced amino acid sequences were examined using the BLAST program (National Center for Biotechnology Information, USA).

### PLASMID ANALYSIS

All *S*. Infantis isolates were examined for the carriage of plasmids by employing the alkaline lysis method as previously described (Kado and Liu, 1981). The molecular size of plasmids was determined by using the standard *Salmonella enterica* serovar Choleraesuis ATCC 7001 (50 kbp) and *S.* Typhimurium DT104 strain 300-98 (90 kbp). The plasmid size was estimated by graphing the molecular size of standard strains versus the distance traveled from the wells using logistic graph paper. Conjugation experiments were performed as described previously (Shahada et al., 2010a). In brief, *S*. Infantis donor isolates and rifampicin-resistant *E. coli* DH5α recipient derivatives were used for conjugal mating. Conjugants were selected onto deoxycholate hydrogen sulfide lactose agar containing 128 μg/ml rifampicin and 128 μg/ml AMP, followed by antimicrobial testing and the detection of transferred resistance genes as above. Plasmids were characterized by the PCR-based replicon typing method as described previously (Carattoli et al., 2005) to detect the plasmid types: IncI1, IncA/C, IncHI1, IncHI2, IncN, IncX, IncW, IncY, IncP, IncT, IncFIIs, IncL/M, IncFIA, IncFIB, IncFIC, IncFrepB, IncK/B, and IncB/O.

## RESULTS

### ANTIMICROBIAL RESISTANCE

*S*. Infantis isolates were recovered in 54 (58.7%) of 92 flocks surveyed and 93 (6.3%) of 1,472 samples examined. Antimicrobial susceptibility profiles of the isolates are summarized in **Table 1**. Of 93 *S*. Infantis isolates recovered, 34 (36.5%) exhibited resistance to AMP (MIC, 256 to >512 μg/ml), 33 (35.5%) showed resistance to CTX (MIC, 4-256 μg/ml) and eight (8.6%) demonstrated resistance to FOX (MIC, 32–128 μg/ml). From the 93 isolates and with regard to non-β-lactam antibiotics, 86 (92.5%) isolates exhibited resistance to STR (MIC, 16–512 μg/ml, conferred by *aadA1*), SUL (MIC >512 μg/ml, conferred by *sul1*) and TET (MIC, 64–512 μg/ml, conferred by* tetA*); 12 (12.9%) isolates showed resistance to KAN (MIC,≥512 μg/ml, conferred by *aphA1*); and 11 (11.8%) isolates demonstrated resistance to OFX (MIC, 2–8 μg/ml). All 93 isolates were fully susceptible to CHL. On the basis of resistance patterns, 11 phenotypes (A to K) were demonstrated as depicted in **Table 2**. The most frequent pattern was STR/SUL/TET (43 isolates) followed by AMP/CTX/STR/SUL/TET (14 isolates).

**Table 1:** Antimicrobial susceptibility among 93 *Salmonella* Infantis isolates detected in this study.

Antimicrobial agent	MIC breakpoint (μg/m/)	No. of resistant isolates (%)	
		This study	Previous study
		2007–2008	2004–2006^a^
AMP	≥32	34 (36.5)	29 (24.2)
CTX	≥4	33 (35.5)	11 (9.1)
FOX	≥32	8 (8.6)	0 (0)
CHL	≥32	0 (0)	0 (0)
STR	≥16	86 (92.5)	120 (100)
SUL	≥512	86 (92.5)	120 (100)
TET	≥16	86 (92.5)	120 (100)
KAN	≥64	12(12.9)	9 (7.5)
OFX	≥2	11(11.8)	25 (20.8)

*CTX, cefotaxime; FOX, cefoxitin; CHL, chloramphenicol; STR, streptomycin; SUL, sulfamethoxazole; TET, oxytetracycline; KAN, kanamycin; OFX, ofloxacin. (a) Cited from the previous study (Shahada et al., 2010b)*

**Table 2:** Distribution of resistance phenotypes, plasmid profiles and β-lactamase phenotypes in *Salmonella* Infantis isolates.

Resistance pattern	Resistance phenotype	Plasmid			No. of isolates	β-lactamase phenotype
		profile	size (ca. kbp)	replicon type
A	AMP/CTX/FOX/STR/SUL/TET/KAN	I	180	IncP	1	AmpC
B	AMP/CTX/FOX/STR/SUL/TET	I	180	IncP	7	AmpC
C	AMP/CTX/STR/SUL/TET/OFX	II	180, 50	IncP, Non-typable	4	ESBL
D	AMP/CTX/STR/SUL/TET/KAN	II	180,50	IncP, Non-typable	2	ESBL
E	AMP/CTX/STR/SUL/TET	I	180	IncP	2	ESBL
	AMP/CTX/STR/SUL/TET	II	180, 50	IncP, Non-typable	11	ESBL
	AMP/CTX/STR/SUL/TET	III	180, 125, 50	IncP, IncA/C, Non-typable	1	ESBL
F	AMP/CTX	II	180, 50	IncP, Non-typable	5	ESBL
G	AMP/STR/SUL/TET	I	180	IncP	1	-
H	STR/SUL/TET/OFX	I	180	IncP	7	-
I	STR/SUL/TET/KAN	I	180	IncP	7	-
J	STR/SUL/TET	I	180	IncP	43	-
K	KAN	I	180	IncP	2	–

*AMP, ampicillin; CTX, cefotaxime; FOX, cefoxitin; CHL, chloramphenicol; STR, streptomycin; SUL, sulfamethoxazole; TET, oxytetracycline; KAN, kanamycin; OFX, ofloxacin*.

### ESBL AND AMPC CHARACTERIZATION

Double-disk synergy testing revealed 25 ESBL- and eight AmpC β-lactamase-producing* S*. Infantis isolates. TEM-type ESBL was identified in 23 (24.7%) isolates, CTX-M-type ESBL was demonstrated in one isolate and CMY-type AmpC β-lactamase was detected in eight isolates. Nucleotide sequencing of PCR products confirmed that the amplicons were *bla*_TEM-52_ (22 isolates), *bla*_TEM-20_ (one isolate), *bla*_CTX-25_ (one isolate in combination with *bla*_TEM-52_), and *bla*_CMY-2_ (8 isolates). Of 54 S. Infantis-positive flocks, As indicated in **Table 3**, *S*. Infantis isolates harboring *bla*_TEM-1_ were not detected during the present study; but, they were found during 1998–2003 (one isolate) and 2004–2006 (17 isolates). On the other hand, isolates harboring *bla*_TEM-52_ were identified during 2004–2006 (11 isolates) and 2007–2008 (22 isolates). Isolates carrying the *bla*_TEM-20_, *bla*_CTX-25__,_ and *bla*_CMY-2_ genes were demonstrated during 2007–2008 study period.

**Table 3:** Chronological change of β-lactamase genes in serovar Infantis.

β-lactamase gene *(bla)*	No. of isolates during the isolation period		
	1998–2003^a^	2004–2006^b^	2007–2008^c^
	(178 isolates)	(120 isolates)	(93 isolates)
TEM-1	1 (0.7%)	17 (14.2%)	0 (0%)
TEM-52	0 (0%)	11 (9.2)	22 (24.4%)^d^
TEM-20	0 (0%)	0 (0%)	1 (1.8%)
CTX-M-25	0 (0%)	0 (0%)	1 (1.8%)
CMY-2	0 (0%)	0 (0%)	8 (8.6%)

a Cited from the previous study (Shahada et al., 2006).

b Cited from the previous study (Shahada et al., 2010b).

c This study.

d CTX-M-25 and TEM-52 were simultaneously identified in one isolate.

### PLASMID CHARACTERIZATION

**FIGURE 1:**
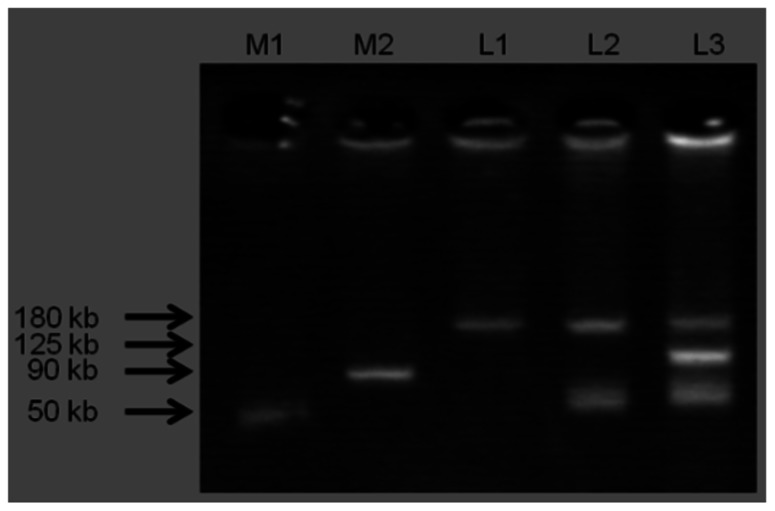
**Three plasmid profiles obtained from *S.* Infantis isolates**. Lane M1 and M2 shows standard sizes of plasmids extracted from *S. Choleraesuis* ATCC 7001 (50 kbp) and *S*. Typhimurium DT104 strain 300-98 (90 kbp), respectively. L1: IncP (ca. 180 kbp), L2: IncP (ca. 180 kbp) and Untypable plasmid (ca. 50 kbp), L3: IncP (ca. 180 kbp), IncA/C (ca. 125 kbp), and Untypable plasmid (ca. 50 kbp).

Three plasmid profiles were defined among *S*. Infantis isolates. Seventy isolates harbored ca. 180-kbp plasmids (profile I), 22 isolates carried two plasmids of ca. 50 and 180 kbp (profile II), and one isolate possessed three plasmids of ca. 50, 125, and 180 kbp (profile III; **Figure 1**). Isolates exhibiting resistance to ESCs displayed diverse profiles: Of 34 ESC-resistant* S*. Infantis isolates, 22 belonged to profile II, 11 were classified into profile I and one isolate was categorized into profile III (**Table 2**). PCR-based replicon typing revealed three kinds of plasmids. Large-size plasmids (ca.180 kbp) were typed as IncP, intermediate-size plasmid (ca. 125 kbp) was typed as IncA/C and small plasmids (ca. 50 kbp) were non-typable. Large-, intermediate- and small-size plasmids were self-transmissible to *E. coli* recipient strain by conjugation. The *bla*_TEM-20_ and *bla*_CMY-2_ genes were associated with large IncP plasmids, *bla*_CTX-M-25_ was affiliated with intermediate IncA/C plasmid and the *bla*_TEM-52_ gene was linked to small non-typable plasmids.

## DISCUSSION

The results obtained in this study show that resistance to ESCs has increased to 35.5% from the previous 9.2% reported during an earlier investigation (Shahada et al., 2010b). Other new findings include the detection of *S. *Infantis isolates harboring the *bla*_TEM-20_, *bla*_CTX-M-25__,_ and *bla*_CMY-2_ genes. Demonstration of these resistance traits in *S*. Infantis serovar is a rare phenomenon. This is the first report describing these mechanisms of ESC-resistance exhibited by *S*. Infantis isolates derived from broilers. Previous reports indicate that β-lactam resistance in broilers was once mediated by *bla*_TEM-1_, a narrow-spectrum β-lactamase gene that was demonstrated in one *S*. Infantis isolate recovered during 1998–2003 (Shahada et al., 2006) and 14.2% of the isolates obtained in 2004–2006 (Shahada et al., 2010a). Besides, during the period of 2004–2006, resistance to ESCs mediated by *bla*_TEM-52_ was reported for the first time from the broiler industry in Japan (Shahada et al., 2010a).

In the present study, we identified one *S*. Infantis isolate harboring the *bla*_TEM-20_ gene while other isolates had *bla*_TEM-52_. It’s worth noting that the wild-type *bla*_TEM-1_ gene was not detected during this study suggesting the likelihood of occurrence of point mutations which led to the emergence of observed variants, *bla*_TEM-20_ and *bla*_TEM-52_. This hypothesis is supported by deduced amino acid sequence analysis which revealed that the *bla*_TEM-20_ gene differs from *bla*_TEM-1_ by two substitutions, Met182→Thr and Gly238→Ser; whereas the *bla*_TEM-52_ gene differs from *bla*_TEM-1_ by three substitutions, Glu104 Lys, Met182→Thr, and Gly238→Ser (Arlet et al., 1999; Weill et al., 2004). This phenomenon has the implications for bacterial adaptation mechanism because *bla*_TEM-1_ seems to have lost its fitness as a potential resistance trait necessary for survival of *Salmonella*. Thus, point mutations have taken place as the evolutionary adaptation mechanism crucial for successful colonization of the broiler chicken intestinal tract.

In Japan, CTX-M-type ESBL-producing *Enterobacteriaceae* are important nosocomial infectious agents raising considerable concern in the public health community. Similarly, the detection of CTX-M-2 and CTX-M-25 ESBL-producing *E. coli* isolates from chickens affected with colibacillosis has raised concerns in the veterinary public health community (Kojima et al., 2005; Asai et al., 2011). We report for the first time one *S*. Infantis isolate harboring *bla*_CTX-M-25_ on IncA/C plasmid and *bla*_TEM-52_ on non-typable plasmid. Because *bla*_CTX-M-25_ was initially demonstrated in *E. coli *(Asai et al., 2011) a comprehensive molecular study is required to determine the likely source and elucidate mechanisms involved in collecting antimicrobial resistance traits and mobilizing them across taxonomical borders.

To date, several reports have described the emergence of AmpC β-lactamase-producing *Salmonella* derived from farm animals affected with salmonellosis. One of the studies involving* Salmonella* *enterica* serovar Typhimurium (*S*. Typhimurium) identified *bla*_CMY-2_ associated with self-transmissible IncI1-Iγ and A/C plasmids (Sugawara et al., 2011). Another study revealed a novel chromosomally integrated multi-drug resistance genomic island harboring *bla*_CMY-2_ among clonally related *S*. Typhimurium isolates (Shahada et al., 2011). Consequently, the detection of *bla*_CMY-2_ in several *S*. Infantis isolates from broilers poses another challenge in the veterinary public health community. Carriers of *bla*_CMY-2_ harbored IncP plasmids initially demonstrated in *Pseudomonas* bacteria (Shintani et al., 2011).

Antimicrobial susceptibility data indicate a gradual decrease of OFX resistance to 11.8 from 20.8% previously reported (Shahada et al., 2010b). Fluoroquinolones (e.g., OFX) are broad-spectrum antimicrobial agents widely used in clinical medicine. Emergence of fluoroquinolone resistance was attributed to overuse of this group of drugs in domestic animals either therapeutically or for the purpose of growth promotion (Asai et al., 2007a). Since 1991, the Japanese Ministry of Agriculture, Forestry and Fisheries (JMAFF) approved this class of antimicrobials in veterinary medicine for therapeutic purposes and prohibited its use as feed additives. Fluoroquinolones might have been prescribed prudently among antimicrobial agents used in broiler farms in Japan. This likely has contributed to the decline of resistance to OFX observed in the present study.

Taken together, these results show increased resistance against ESCs mediated by both ESBLs and AmpC β-lactamases. It seems these resistance traits have spread among farm animals in Japan while their likely source remains largely unknown. The probability that resistance to ESCs may continue to spread among members of the *Enterobacteriaceae* family poses another public health challenge because ESBLs and AmpC β-lactamases limit the effectiveness of cephalosporin therapy.

## Conflict of Interest Statement

The authors declare that the research was conducted in the absence of any commercial or financial relationships that could be construed as a potential conflict of interest.
